# Evidence of increasing sedentarism in Mexico City during the last decade: Sitting time prevalence, trends, and associations with obesity and diabetes

**DOI:** 10.1371/journal.pone.0188518

**Published:** 2017-12-01

**Authors:** Catalina Medina, Lizbeth Tolentino-Mayo, Ruy López-Ridaura, Simón Barquera

**Affiliations:** Mexican National Institute of Public Health, Cuernavaca, Morelos, México; Vanderbilt University, UNITED STATES

## Abstract

**Introduction:**

Sedentary behaviors such as sitting time are associated with obesity and diabetes independently of total reported physical activity. This study aimed to describe the current sitting time/day prevalence and trends and to examine the association of sitting time with sociodemographic and clinical variables in Mexico City.

**Methods:**

Two cross-sectional representative surveys in Mexico City were used for this analysis (2006: n = 1148 and 2015: n = 1329). Sedentary behavior questions from the International Physical Activity Questionnaire included time spent sitting on a weekday in the last week or on a Wednesday. Sitting time /day was divided into deciles, and participants in the highest decile (≥ 420 minutes/day) were classified within the high sitting category; others were classified in the low sitting time category. Multivariate logistic regression was used to evaluate the associations of sitting time with sociodemographic and clinical indicators, controlling for confounders and testing for potential interactions.

**Results:**

A total of 13.7% (2006) and 14.8% (2015) adults were classified in the highest sitting time category (≥ 420 minutes/day). There was a significant increase in the average sitting time/day between the surveys **(**216.0 minutes in 2006 vs. 233.3 minutes in 2015, p < 0.001). In 2015, men, those aged 20–49 years, those in low-intensity jobs, students, and those with a high socioeconomic level were more likely to be in the highest sitting time category. Participants with overweight/obesity (OR = 2.37, 95% CI: 1.11, 5.09) and those with high glucose levels (survey finding) (OR = 2.34, 95% CI: 1.04, 5.25) were more likely to report sitting time in the highest category.

**Discussion:**

Sitting time/day prevalence increased 8%, and average daily sitting minutes significantly increased by 8.2% (18 minutes) in the nine-year study period (2006–2015). Current public health policies should consider strategies not only for increasing physical activity levels, but also for reducing sitting time/day among the population as a measure to fight the growing epidemic of obesity and diabetes in Mexico.

## Introduction

Cardiovascular diseases, type 2 diabetes (T2D), respiratory diseases, and cancer are the four major public health problems worldwide [1]. According to the World Health Organization (WHO), 38 million deaths due to Non-Communicable Diseases (NCDs) occurred in 2012, and 82% of these deaths in low- and middle-income countries [1]. Among the main modifiable risk factors associated with this burden, three of the most recognized are 1) the lack of physical activity; 2) unhealthy diets; and 3) sedentary behaviors such as time spent in front of a screen, inactive transportation, sitting-based activities, and domestic entertainment technology [2].

Sedentary behaviors are those in which energy expenditure is ≤ 1.5 metabolic equivalent units (METs) [3]. Recent studies have reported that the benefits of being physically active may be compromised as more hours are spent on sedentary activities [4]. Furthermore, prolonged sedentary behavior is associated with metabolic syndrome and obesity [4–7], which, in turn, are associated with increases in T2D and cardiovascular diseases [8].

In the last decade, the importance of measuring sedentary behaviors on population-based surveys has increased. These behaviors have been measured in some countries using either the International Physical Activity Questionnaire (IPAQ) [9,10] or the Global Physical Activity Questionnaire [11,12]. In an international study of the prevalence of sitting time, Portugal, Brazil, and Colombia had a relatively low amount of sitting time, in contrast to Taiwan, Norway, Hong Kong, Saudi Arabia, and Japan, which reported some of the longest average sitting time [13]. To date, there has been no report describing sitting time prevalence in Mexico based on data from a representative sample.

Given the high prevalence and alarming increase in obesity, diabetes, and other NCDs observed in Mexico, sedentary behavior patterns and their contribution to potential risk factors need to be understood, particularly in Mexico City, the largest city in Latin America, where urbanization and sedentary lifestyles coexist with poor quality diets [14]. Thus, our study aimed to describe the current prevalence and recent trends in sitting time and to examine the association between sitting time and sociodemographic characteristics and clinical indicators in adults (aged 20–69 years) in Mexico City.

## Materials and methods

This study was designed to estimate the sitting time prevalence and trends using data from two representative surveys, conducted in 2006 and 2015. In a second stage, the last survey (2015) was used to estimate the association of average daily sitting time with sociodemographic characteristics and clinical indicators in adults (aged 20–69 years) in Mexico City.

### Design and participants

For our analysis, we used data from the Mexican Nutrition and Health Survey (ENSANUT, its acronym in Spanish) 2006, collected from October 2005 to May 2006, and the Mexico City Diabetes Representative Survey (MCDRS) 2015, collected from May to June 2015. These surveys have a probabilistic multistage stratified cluster sampling design and share the same basic methodology for the collected variables [15,16]. We used the ENSANUT 2006 Mexico City subsample and the MCDRS 2015 sample of adults (aged 20–69 years) who completed the IPAQ (2006: n = 1148 and 2015: n = 1319). Additional details of the methodology used to obtain these survey data have been published elsewhere [15,16].

### Sitting time assessment

Sitting time was assessed on both surveys by asking two items from the Spanish version of the IPAQ short form [10,17]: 1) “During the last seven days, how much time did you usually spend sitting on a weekday?” or 2) “During the last seven days, how much time did you usually spend sitting on a Wednesday?” Participants who reported > 960 minutes/day (> 16 hours/day) or < 10 minutes/day of sitting were excluded from the analysis, following the IPAQ methodology cut points [18]. The time spent sitting per day reported either on a weekday or on a Wednesday was divided into deciles. Respondents in the highest decile (≥ 420 minutes/day) were categorized as having high sitting time; others were categorized as having low sitting time. These questions have been previously validated using accelerometers (r = 0.34) [9].

### Covariates used in the description of sitting time prevalence and trends in 2006 and 2015

#### Socioeconomic status and education

For both surveys, a socioeconomic status (SES) index was constructed by combining eight variables that assessed household characteristics, goods, and available services, including construction materials of the floor, ceiling, and walls; household goods (stove, microwave, washing machine, refrigerator, and boiler); and electrical goods (television, computer, radio, and telephone). The index was divided into tertiles and used as a proxy for low, medium, and high SES [19]. Education level was categorized into three groups according to the highest level of education obtained: primary or less, secondary, and high school or higher.

#### Anthropometric measurements

On both surveys, weight and height were measured to the nearest 0.1 kg and 0.1 cm, and body mass index (BMI) was calculated as kg/m^2^. BMI status was based on the WHO’s adult cut points: underweight (< 18.5 kg/m^2^), normal weight (18.5–24.9 kg/m^2^), overweight (25.0–29.9 kg/m^2^), and obese (≥ 30.0 kg/m^2^) [20]. For the present study, BMI was divided into two categories: normal weight (< 24.9 kg/m^2^) and overweight/obese (≥ 25.0 kg/m^2^).

#### Diabetes status

Participants were classified as 1) non-diabetics; 2) having survey findings of fasting glucose (at least 8 hours) ≥ 7.0 mmol/L (126 mg/dL) without a previous medical diagnosis; or 3) previously diagnosed, if participants self-reported a previous medical diagnosis of diabetes [21].

#### Total energy intake

Standardized personnel administered a food frequency questionnaire that asked about respondents’ dietary intake over the previous seven days, including portion sizes of the foods consumed. This questionnaire included 126 foods from 14 different food groups and was the same instrument used on the Mexican National Health and Nutrition Survey. Dietary intake data were converted to average grams or milliliters per person per day. A nutritional composition database compiled by the Mexican National Institute of Public Health was used to estimate the total energy intake per day [22].

### Sociodemographic and clinical covariates from the MCDRS 2015 tested for associations with sitting time

#### Hemoglobin A1c (HbA1c)- 2015

Trained personnel collected blood samples from the antecubital vein. Blood samples were centrifuged at the site of collection. Aliquots of blood were transported in ice chests with liquid nitrogen and stored at −70° C until they were analyzed. Glycosylated hemoglobin was processed in Bio-Rad Variant II turbo by means of high-pressure liquid chromatography.

#### Family history of diabetes in 2015

Family history of diabetes was classified as “yes” or “no,” based on the response to the following question: “Do your parents have increased levels of glucose?”

#### Waist circumference in 2015

Waist circumference was measured twice by trained personnel to the nearest 0.1 cm at the narrowest point between lower border of the rib cage and iliac crest using a fiberglass tape measure (Gülick). The average waist circumference was used for further analysis.

#### Perceived occupational physical activity intensity in 2015

Perceived occupational physical activity intensity was assessed using the following question: “How active or heavy do you feel your main job is?” Answers were classified into two categories: 1) light and 2) moderate/vigorous.

#### Current employment status in 2015

Participants were asked “What is your current working status?” Answers were classified into four categories: 1) housewife/unemployed; 2) working; 3) retired; and 4) student. In total, 26 cases were excluded from this variable because respondents did not specify their current working status, and two respondents reported a physical disability.

#### Type of job (METs) in 2015

Participants were asked to describe their main job. Types of jobs were categorized according to the 2002 census occupational classification system [23]. A MET value was assigned to each category based on the compendium of occupational activities [24]. Household activities were also classified, taking this system into account, using the average value of the different household tasks (light, moderate, and vigorous) as a proxy [25]. The resulting continuous MET values were stratified into tertiles (1.5 to 2.4, 2.5 to 3.2, and 3.3 or higher). In some cases, it was not possible to derive the MET value from the reported occupation (n = 25) or respondents said that they were retired and did not describe their activities (n = 37); these cases were not considered for this variable.

#### Consent to participate

All participants provided informed consent prior to participating (No. B04). The Ethics Review Board of the Mexican National Institute of Public Health approved both study protocols (No.1658).

### Statistical analysis

Percentages, means, and 95% confidence intervals (95% CI) were used to describe the sitting time prevalence and the sociodemographic, clinical, and dietary factors of the 2006 and 2015 survey samples.

To evaluate the effect of population structure, average minutes/day of sitting was age- and sex-standardized, using 2015 as the reference. This procedure generated a 0.4 minutes/day increase, which was not considered a relevant change, considering the wide interval range. Thus, the means and prevalence values for both surveys are presented as non-standardized values.

We evaluated the distribution of the average sitting time/day using the Kolmogorov–Smirnov test. Because of the lack of normality, average sitting time/day was log-transformed prior to subsequent analyses. We used complex sample general linear models to compare the log-transformed mean of sitting time/day by sociodemographic factors (gender, age, BMI, SES, educational level, and diabetes status) and by survey year (2006 vs. 2015). Kernel-density plots were calculated to visualize the proportional amount and the distribution of mean sitting time/day by survey (2006 vs. 2015).

Differences in the prevalence of sitting time/day by sociodemographic, anthropometric, and clinical factors were tested using the chi-square test. Univariate logistic regression (crude model) was used to evaluate the association between the highest level of sitting time (≥ 420 minutes/day) and sociodemographic, anthropometric, dietary, and clinical indicators. We created five multivariate logistic regression models: Model 1 adjusted for sex, age (20 to 49 or 50 to 69), and BMI (normal weight or overweight/obese); Model 2 adjusted for the variables used in Model 1 and previously diagnosed diabetes (yes, no, or survey findings); Model 3 adjusted for the variables used in Model 2, plus perceived occupational intensity (moderate/vigorous or light) and educational level (primary or less, secondary, or high school or higher); Model 4 adjusted for the variables used in Model 3, type of job in METs (1.5 to 2.4, 2.5 to 3.2, or 3.3 or higher), total energy intake, and family history of diabetes; and Model 5 adjusted for the variables used in Model 3, current employment status (housewife/unemployed, working, retired, or student), total energy intake, family history of diabetes, HbA1C values, and waist circumference. These models were tested for multicollinearity. The level of significance was set at p < 0.05 for the regression model estimates.

We tested interactions between 1) age and perceived physical activity at work (interaction not significant) and 2) body mass index and perceived physical activity at work in terms of their association with sitting time, using a multivariate logistic regression adjusting for potential confounders. The level of significance was set at p < 0.1 for the interactions.

Statistical analyses were conducted using SPSS version 24 for complex samples (IBM SPSS statistics, IBM Corporation, Somers, NY). Calculations were adjusted by sample weights and by cluster and strata variables.

## Results

Of the 1158 participants in 2006 and 1334 in 2015, we excluded pregnant women (n = 12 in 2006 and n = 8 in 2015), those who could not stand (n = 157 and n = 7 in 2006 and 2015, respectively), and those who reported < 10 minutes of sitting/time per day (n = 14 in 2006 and n = 8 in 2015), leaving a final sample size of 970 in 2006 and 1312 in 2015.

The sociodemographic and anthropometric characteristics of adults aged 20–69 years in Mexico City in 2006 and 2015 are described in Table 1. In both years, more than 70% of the sample was aged < 50 years. Overweight/obesity increased 5.4% over the nine-year study period, from 70.2% in 2006 (95% CI: 65.5, 74.5) to 74.0% in 2015 (95% CI: 70.3, 77.3). The median total energy intake was 1715 (IQR: 1250, 2224) kcal/day for 2006 and 2152 (95% CI: 1554, 3049) kcal/day for 2015. In total, 8.8% of the respondents had a previous diagnosis of diabetes in 2006, and this rose to 10.1% in 2015. The prevalence of participants being classified in the highest sitting time category (≥ 420 minutes/day) increased by 8% (from 13.7% in 2006 to 14.8% 2015).

**Table 1:** Sociodemographic and anthropometric characteristics of the analytic sample of adults aged 20–69 years in Mexico City (ENSANUT 2006 and MCDRS 2015).

	2006 n = 970 N = 4,550,965	2015 n = 1,312 N = 5,417,084
	% (95% CI) or median (IQR)	% (95% CI) or median (IQR)
**Gender (%)**		
Men	49.4 (46.4, 52.5)	47.3 (44.6, 50.0)
**Age group (%)**		
20–49	75.5 (71.4, 79.2)	74.4 (71.9, 6.8)
50–69	24.5 (20.8, 28.6)	25.6 (23.2, 28.1)
**BMI Classification (%)**		
Normal weight	29.8 (25.5, 34.4)	26.0 (22.7, 29.7)
Overweight/Obese	70.2 (65.5, 74.5)	74.0 (70.3, 77.3)
**Socioeconomic status (%)**		
Low	23.5 (19.3, 28.2)	22.2 (19.0, 25.8)
Medium	30.4 (27.1, 34.0)	36.6 (32.8, 40.5)
High	46.1 (40.2, 52.0)	41.2 (36.6, 46.3)
**Educational level (%)**		
Primary or less	23.4 (20.0, 27.1)	19.2 (16.4, 22.3)
Secondary	26.4 (22.5, 30.6)	28.0 (24.5, 31.7)
High school or higher	50.2 (44.3, 56.1)	52.9 (48.7, 57.0)
**Sitting time (%)**		
<420 min/day	86.3 (83.1, 89.0)	85.2 (82.3, 87.7)
≥420 min/day	13.7 (11.0, 16.9)	14.8 (12.3, 17.7)
**Total energy intake (median)**		
(Kcal/day)	1715 (1250, 2224)	2152 (1554, 3049)
**Diabetes (%)**		
Previously diagnosed	8.8 (5.2, 14.5)	10.1 (8.8, 11.5)
No	79.4 (72.2, 85.1)	85.9 (83.9, 87.7)
Survey finding	11.8 (7.4, 18.3)	4.0 (3.1, 5.2)

% (95% CI) = prevalence (95% confidence interval).

ENSANUT: Mexican Nutrition and Health Survey.

MCDRS: Mexico City Diabetes Representative Survey.

Table 2 shows the mean and median sitting time and trends for adults living in Mexico City by diverse sociodemographic factors. Overall, sitting time increased 17.3 minutes on average from 2006 to 2015. A significant increasing trend was observed from 2006 to 2015 among women, adults aged 20–49 years, adults with normal BMIs, overweight/obese adults, those in low and medium SES categories, those with primary and secondary educational levels, those who had previously been diagnosed with diabetes, and those who did not have a diabetes diagnosis or survey findings.

**Table 2:** Sitting time (minutes/day) trends among adults aged 20–69 years in Mexico City by sociodemographic factors^♦^.

	ENSANUT 2006		MCRDS 2015		
Sociodemographic factors	Mean (95%CI)	Median	Mean (95% CI)	Median	P value^X^
**Total**	216.0 (202.9, 229.2)	180	233.3 (222.1, 244.5)	180	0.001*
**Gender**					
Males	232.1 (208.9, 255.2) ^b^	180	251.9 (236.1, 267.7) ^b^	240	0.054
Females	200.3 (186.4, 214.2)	180	216.6 (203.7, 229.5)	180	<0.001*
**Age group**					
20–49	221.1 (205.3, 237.0)	180	243.3 (229.9, 256.6) ^b^	180	0.001*
50–69	200.2 (176.9, 223.4)	180	204.3 (188.4, 220.2)	180	0.326
**BMI Classification**					
Normal weight	185.7 (158.0, 213.3)	120	226.5 (210.6, 242.4)	180	0.001*
Overweight/obese	218.5 (197.0, 240.0)	180	235.2 (219.7, 250.8)	180	0.045*
**Socioeconomic status**					
Low	175.5 (152.8, 198.2)	120	210.3 (194.7, 225.9)	180	0.001*
Medium	196.3 (179.7, 212.8)^a^^,^^c^	180	221.5 (206.6, 236.4) ^a^^,^^c^	180	0.027*
High	249.7 (231.0, 268.3) ^a^^,^^b^	180	256.2 (234.1, 278.3) ^a^^,^^b^	240	0.135
**Educational level**					
Primary or less	164.7 (145.7, 183.7)	120	183.0 (165.0, 201.1)	150	0.023*
Secondary	197.2 (171.5, 222.9) ^a^^,^^c^	120	220.0 (204.4, 235.6) ^a^^,^^c^	180	0.030*
High school or higher	250.0 (234.1, 267.9) ^a^^,^^b^	180	258.6 (242.1, 275.0) ^a^^,^^b^	240	0.155
**Diabetes**					
Previously diagnosed	167.8 (102.3, 233.3)	120	214.9 (197.0, 232.9)	180	0.016*
No	209.7 (175.0, 244.3)	180	235.9 (223.4, 248.5)	180	0.022*
Survey findings	273.9 (187.9, 359.9) ^a^	180	223.4 (182.1, 264.7)	180	0.331

^***X***^ Log-transformed p trend (2006 vs. 2015).

^**♦**^ ENSANUT: Mexican Nutrition and Health Survey 2006, MCDRS: Mexico City Diabetes Representative Survey 2015.

a,b,c Different superscripted letters represent log-transformed statistically significant differences between categories (p < 0.05).

* Statistically significant differences between 2006 and 2015.

**Fig 1:**
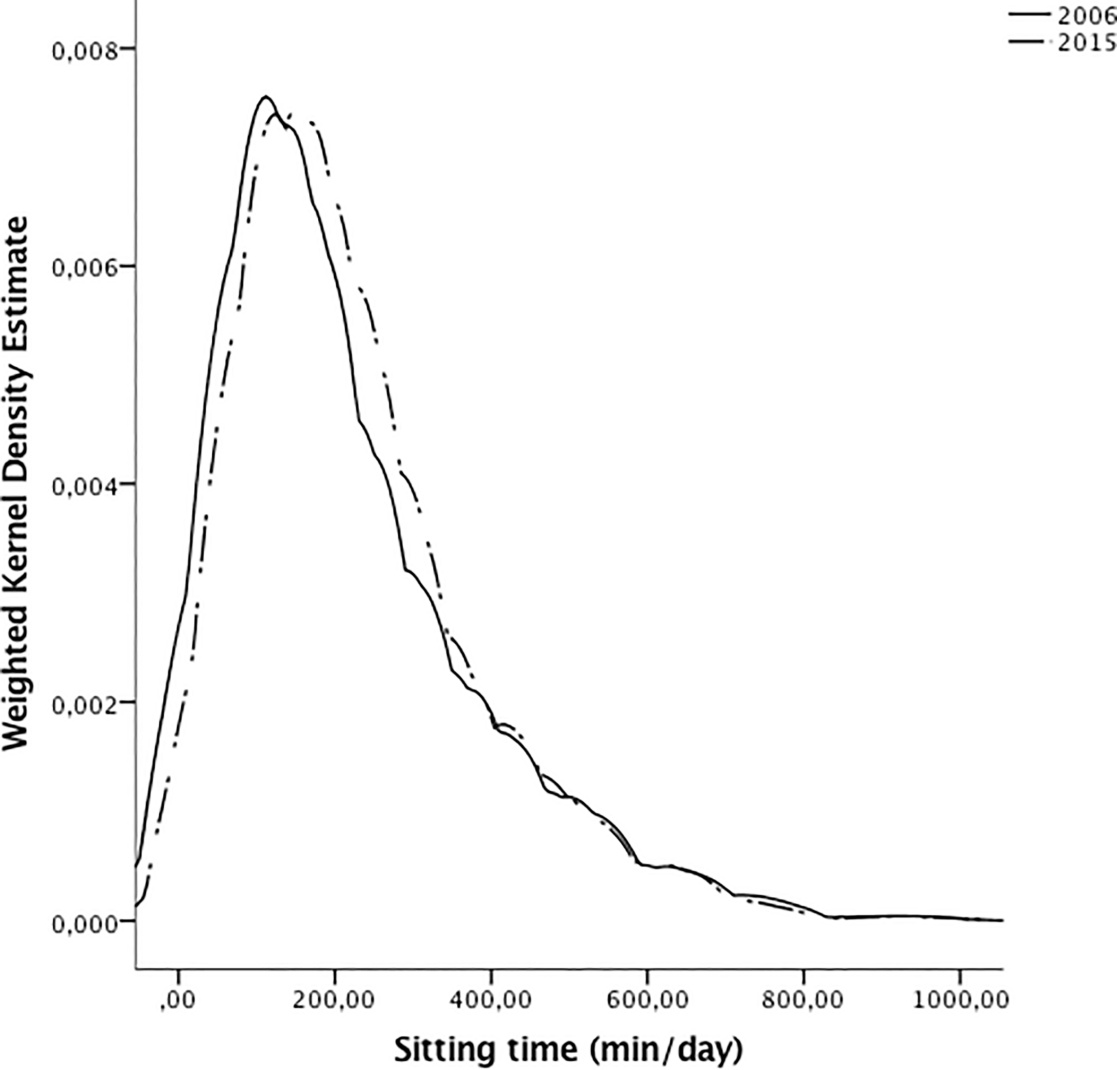
Sitting time (minutes/day) trend among adults aged 20–69 years in Mexico City. Mexican Nutrition and Health Survey 2006 and Mexico City Diabetes Representative Survey 2015.

The distribution of sitting time/day from 2006 to 2015 is graphically presented in Fig 1, where a slight shift to the right is observed for 2015, reflecting an increase in the minutes spent sitting per day in the overall the population.

The prevalence of sitting time by sociodemographic and anthropometric characteristics in 2015 is shown in Table 3. Men (19.8%), those aged 20–49 years (16.4%), the overweigh/obese group (16.4%), those with high levels of glucose (survey finding) (21.4%), those who perceived their jobs to be low intensity (27.3%), housewives/unemployed (16.3%), students (39.9%), those in jobs with lower energy demands (25.1%), and those with a higher educational level (18.2%) had a significantly higher prevalence of high sitting time (≥ 420 minutes/day), compared with their counterparts.

**Table 3:** Crude and adjusted odds ratios (OR) of being in the highest sitting category (≥ 420 minutes/day), MCDRS, Mexico City, 2015.

Independent variables	Sitting time (≥420 min/day) % (95% CI)	Crude model OR (95% CI)	Model 1 OR (95% CI) N = 1300	Model 2 OR (95% CI) N = 1300	Model 3 OR (95% CI) N = 1245	Model 4 OR (95% CI) N = 1195	Model 5 OR (95% CI) N = 1196
**Gender**							
Women	10.4 (8.2, 13.1)	1	1	1	1	1	1
Men	19.8 (15.7, 24.8) ^a^	**2.18 (1.53, 3.13)**	**2.25 (1.56, 3.23)**	**2.67 (1.86, 3.82)**	**2.27 (1.61, 3.19)**	**2.08 (1.36, 3.16)**	**1.99 (1.21, 3.27)**
**Age group**							
50–69	10.2 (7.4, 14.0)	1	1	1	1	1	1
20–49	16.4 (13.5, 19.9) ^a^	**1.76 (1.18, 2.62)**	**1.79 (1.18, 2.73)**	**1.77 (1.14, 2.75)**	**1.71 (1.07, 2.75)**	**1.98 (1.15, 3.39)**	**1.85 (1.01, 3.39)**
**Body mass index**							
Normal	9.8 (6.7, 14.2)	1	1	1	1	1	1
Overweight/obese	16.4 (13.2, 20.2) ^a^	**1.80 (1.06, 3.05)**	**1.98 (1.13, 3.45)**	**1.95 (1.10, 3.44)**	**2.89 (1.53, 5.45)**	**2.57 (1.34, 4.95)**	**2.37 (1.11, 5.09)**
**Diabetes**							
Previously diagnosed	10.7 (7.0, 15.9)	1	-	1	1	1	1
No	15.0 (12.2, 18.3)	1.47 (0.85, 2.53)	-	1.17 (0.65, 2.09)	1.09 (0.60, 2.01)	1.20 (0.63, 2.28)	1.40 (0.60, 3.25)
Survey finding	21.4 (14.4, 30.7) ^a^	**2.28 (1.12, 4.62)**		1.76 (0.85, 3.63)	1.92 (0.89, 4.14)	**2.14 (1.00, 4.59)**	**2.34 (1.04, 5.25)**
**Perceived occupational intensity**			-				
Moderate/vigorous	11.0 (8.9, 13.6)	1		-	1	1	1
Light	27.3 (20.7, 35.2) ^a^	**3.20 (2.14, 4.77)**	-	**-**	**3.49 (2.27, 5.38)**	**2.94 (1.70, 5.07)**	**3.11 (1.90, 5.09)**
**Current working status**			**-**				
Housewife/unemployed	16.3 (13.7, 19.4) ^b^^,^^c^^,^^d^	1		-	-	-	1
Working	7.1 (4.1, 12.1)	**2.77 (1.38, 5.55)**	-	**-**	**-**	**-**	**1.95 (0.87, 4.36)**
Retired	18.5 (7.8, 37.6)	**2.73 (1.02, 7.29)**	**-**	-	-	-	3.13 (0.70, 14.0)
Student	39.9 (22.2, 60.7) ^b^^,^^c^	**10.63 (3.71, 30.4)**	-	**-**	**-**	**-**	**8.07 (2.39, 27.3)**
**Type of job (METs)**			**-**				
3.3 +	7.8 (5.1, 11.9)	1		-	-	1	-
2.5–3.2	8.7 (5.8, 12.8)	1.19 (0.71, 2.02)	-	-	-	0.96 (0.56, 1.63)	-
1.5–2.4	25.1 (20.4, 30.5) ^a^^,^^b^	**4.30 (2.37, 7.82)**	-	**-**	**-**	**2.80 (1.50, 5.22)**	**-**
**SES**			**-**				
Low	13.6 (10.4, 17.5)	1		-	-	-	-
Medium	12.5 (9.6, 16.2)	0.98 (0.64, 1.49)	-	-	-	-	-
High	17.5 (12.5, 24.1)	1.48 (0.85, 2.56)	-	-	-	-	-
**Educational level**			-				
Primary or less	10.0 (5.7, 16.8)	1		-	1	1	1
Secondary	11.8 (8.6, 16.0)	1.29 (0.61, 2.71)	-	-	1.15 (0.55, 2.42)	0.99 (0.41, 2.40)	1.07 (0.46, 2.45)
High school or higher	18.2 (14.3, 23.0) ^a^^,^^b^	**2.11 (1.05, 4.24)**	-	**-**	**2.11 (1.07, 4.16)**	1.68 (0.82, 3.47)	**1.70 (0.82, 3.51)**

MCDRS: Mexico City Diabetes Representative Survey 2015.

Model 4 adjusted for total energy intake and family history of diabetes.

Model 5 adjusted for total energy intake, family history of diabetes, HbA1C values, and waist circumference.

a,b,c,d Different superscripted letters represent statistically significant differences between categories (p < 0.05).

To explore associations with the highest sitting time category, we tested diverse multivariate logistic regression models (Table 3). Men (vs. women), those aged 20–49 years (vs. those aged ≥ 50), overweight/obese people (vs. normal-weight), those with high levels of glucose (survey finding) (vs. non-diabetics and those previously diagnosed with diabetes), those with jobs perceived to be low intensity (vs. moderate/vigorous), students and those who are employed (vs. the housewives/unemployed category), and those with the highest level of education (vs. primary or less and secondary) were significantly more likely to be in the highest sitting time category (≥ 420 minutes/day), after adjusting for the covariates described above.

Adults who reported a perception of light physical activity at work had higher mean BMI (28.9 vs. 27.8, p = 0.015) and higher mean minutes/day of sitting (285.0 vs. 217.7, p < 0.001), compared with those who reported a perception of moderate/vigorous physical activity at work. After adjusting for age, sex, diabetes, current employment status, and educational level, the interaction between BMI and perceived physical activity at work in terms of the association with sitting time was significant (p = 0.011).

## Discussion

The present study has shown that the prevalence of high sitting time (≥ 420 minutes/day) increased by 8% over nine years (from 13.7% in 2006 to 14.8% in 2015) in Mexico City. In addition, a significant average increase of 17.3 minutes/day in sitting time was observed from 2006 to 2015 in Mexico City. This increase was significantly higher among women, those aged 20–49 years, those who are normal weight, the overweight/obese group, people with low or medium SES, those with primary or secondary education levels, those who were previously diagnosed with diabetes, and non-diabetic individuals. Finally, based on the 2015 survey, men, those aged 20–49 years, overweight/obese individuals, participants with high levels of glucose (survey finding), those who perceived their jobs to be low intensity, students, working people, and participants with the highest educational level were more likely to be in the highest sitting category (≥ 420 minutes/day).

Whereas a previous study of 17 European countries from 2002 to 2013 [26] documented a decline in sitting time, our study in Mexico showed a significant increase from 2006 to 2015 using the IPAQ and representative surveys. Although Mexico City is one of the most highly populated cities in the world, sitting time/day reported on both surveys (216.0 minutes in 2006 and 233.3 minutes in 2015) was slightly lower than the estimated sitting times for other highly urbanized cities and is similar to levels reports by other developing countries. For instance, a study that estimated sitting prevalence among the German adult population reported an average sitting time of 316.7 minutes/day [27]. A study describing the average sitting time/day in 20 countries reported that Saudi Arabia, Japan, Taiwan, Norway, Lithuania, Hong Kong, and the Czech Republic had the highest median values (≥ 360 minutes/day), whereas Latin American countries (Colombia and Brazil) had the lowest median values (180 minutes/day) [13]. Moreover, a study of 32 European countries revealed that Romania and Portugal had the lowest mean values of sitting time/day (191 minutes and 194 minutes, respectively), in contrast to the Netherlands, Denmark, the Czech Republic, and Greece, which had mean sitting times > 360 minutes/day [28]. Finally, a study from 28 European Union member states found that the median minutes of sitting/day was 300, with countries’ values ranging from a low of 180 minutes in Portugal to a high of 360 minutes in Denmark and in the Netherlands. Possible explanations for these differences could be associated with income, development, and educational levels. Our study showed that people in the highest educational and socioeconomic categories spent significantly more minutes/day performing sitting time activities than did those in the lowest categories; however, mean values found in this study are lower (< 260 minutes/day) than those reported for developed countries (> 360 minutes/day) [26]. This is consistent with the conclusion reached from other studies: Individuals living in developing countries may sit less and have higher energy demands from their occupations, compared with those in developed countries [13,29].

Based on the 2015 data, we observed that men, compared with women, spent more minutes sitting per day and were approximately twice as likely to be in the highest category of sitting per day [13,28]. A study conducted in 32 countries showed that women spent less minutes sitting per day and were less likely to be in the highest sitting deciles, compared with men [28]. Although their finding was not significant, Bauman et al. [13] observed the same result in a study performed in 20 countries. An explanation for the lower levels of sitting time/day among women could be that they spend most of their daily time engaged in low-intensity activities, such as family-care activities, workday activities, and/or domestic duties.

In addition, in this study, those in the housewives/unemployed category (mostly women) were less likely to be in the highest sitting deciles, compared with other employment categories (working, retired, and students). In line with this, Brown et al. [30], who explored the relationship between sitting time and BMI, reported that Australian women engaged in full-time home duties reported significantly lower overall sitting time than did full-time workers. In addition, a study performed in India that described the physical activity levels of urban middle-age women in different domains found that women’s maximum energy expenditure was in household chores and their minimum was in recreational exercise and transportation [31]. This pattern could explain why women have lower levels of sitting and lower levels of moderate/vigorous physical activity. Future studies should more deeply explore the benefits of light physical activities (mostly activities performed at home) on health, not only for the potential contribution to reducing sitting time, but also to inform public health recommendations.

Participants in the older group were less likely to report the highest category of sitting time/day. This is consistent with age differences reported previously [13,27,28]. Some explanations of these results could be related to a self-report bias or an increase in recreational and household activities [32] among the oldest group, in contrast to the youngest group’s use of motorized vehicles and technology (television, smartphones, tablets and computers) and their school-related activities. In line with this result, the present study has shown that students aged 20–31 years were more likely to be in the highest sitting category, compared with the current housewives/unemployed category.

Overweight and obese participants were more likely to report the highest level of sitting time in Mexico City, even after adjusting for waist circumference, and this result is similar to the findings of previous studies [2,33–35]. However, a study performed using a representative sample in Germany with the main objective of determining the sociodemographic and environmental correlates of sitting reported contradictory findings [27]. Among the reasons the authors reported were the limited evidence on the association between overall sitting and weight gain and the use of a self-reported BMI, which could have resulted in misclassification.

Our results are in line with those reported in previous studies finding that, after controlling for potential confounders, individuals reporting jobs with low-energy demands (i.e., office and administrative support occupations, students) were more likely to be in the highest sitting category, compared with those with jobs with high energy demands. Some authors have suggested strategies (i.e., sit-to-stand workstations, take a stand interventions) to reduce sitting time among jobs with low energy demands [28,36–41]. However, these strategies have the disadvantage of compensating sitting time outside work, being ineffective in the long term, and being expensive [40,41]. Further effective desk-based interventions are needed to reduce not only sitting time at work, but also during leisure time.

Participants with a perception of light physical activity at work were significantly more likely to be in the highest sitting level category (≥ 420 minutes/day), compared with those with a perception of moderate/vigorous physical activity at work. The test of the interaction between perceived intensity of physical activity at work and BMI revealed those individuals who perceived light physical activity at work had higher BMI and higher minutes of sitting/day, compared with those who perceived moderate/vigorous physical activity at work. Considering the cause–effect limitation of this study, these results may indicate that higher sitting time at work increase BMI levels. However, the relationship between sitting at work and BMI is still inconsistent with the findings of previous studies [30,40,42,43].

Finally, people with high levels of blood glucose (survey finding) were more likely to be in the highest sitting category (≥ 420 minutes/day) than were those with a previous diagnosis of diabetes. Total sitting time and its association with diabetes has been studied in previous cross-sectional [44,45], cohort [44,46], and intervention studies [44], with inconsistent results [47]. The lack of consistency among the evidence could be related to the limited information on minutes of sitting stratified by newly and previously diagnosed diabetes, the use of self-report questionnaires to measure sitting time [26,45], the attenuating effect of adiposity on the association between sitting and biomarkers of metabolic health [44,45], the use of total sitting instead of domain-specific sitting in the association with diabetes [45], and differences in the effect of sitting by gender [45].

Although this study is limited by the use of a self-report instrument, sitting time minutes and prevalence were obtained in both survey years using the same items from the short-form IPAQ. In addition, although the IPAQ was designed and validated for use in population surveillance, this instrument, like other questionnaires, is subject to recall and social desirability bias [48]. Previous studies have demonstrated that participants underestimate their sitting time, and this misclassification of sitting time could have attenuated the association between sitting and diabetes [26]. Although we used the cut points suggested by the IPAQ team, this criterion could have influenced the effect of sitting on health outcomes. Because research of sedentarism is a relatively new area, the cut-off points and the use of objective methods to evaluate sitting time are still becoming more precise and accessible. In the near future, these developments will allow improvements in data quality. Finally, causality should not be inferred from the present study. Future studies should consider the inclusion of variables that evaluate sitting time on weekdays, weekends, and across different domains, such as work, transportation, home, and leisure, to understand the impact of these sedentary domains on health conditions.

## Conclusions

Our study found that the prevalence of sitting time/day increased from 13.7% to 14.8%, and the average sitting time/day increased 17.3 minutes from 2006 to 2015 in Mexico City. Sitting time was associated with overweight/obesity and with high levels of glucose (survey finding). This study highlights the importance of measuring the prevalence and trend in sedentary behaviors on a national survey. Current public health policies should consider strategies for reducing sitting time/day among the population as a measure to fight the growing epidemic of obesity and diabetes in Mexico. Futures studies should explore the health impact of different sitting domains (i.e., work/school, transportation, home, and leisure) to allow for more focused public health recommendations.
